# Compliance-Free, Digital SET and Analog RESET Synaptic Characteristics of Sub-Tantalum Oxide Based Neuromorphic Device

**DOI:** 10.1038/s41598-018-19575-9

**Published:** 2018-01-19

**Authors:** Yawar Abbas, Yu-Rim Jeon, Andrey Sergeevich Sokolov, Sohyeon Kim, Boncheol Ku, Changhwan Choi

**Affiliations:** Division of Materials Science and Engineering, Hanyang University, Seoul, 04763 Republic of Korea

## Abstract

A two terminal semiconducting device like a memristor is indispensable to emulate the function of synapse in the working memory. The analog switching characteristics of memristor play a vital role in the emulation of biological synapses. The application of consecutive voltage sweeps or pulses (action potentials) changes the conductivity of the memristor which is considered as the fundamental cause of the synaptic plasticity. In this study, a neuromorphic device using an *in-situ* growth of sub-tantalum oxide switching layer is fabricated, which exhibits the digital SET and analog RESET switching with an electroforming process without any compliance current (compliance free). The process of electroforming and SET is observed at the positive sweeps of +2.4 V and +0.86 V, respectively, while multilevel RESET is observed with the consecutive negative sweeps in the range of 0 V to −1.2 V. The movement of oxygen vacancies and gradual change in the anatomy of the filament is attributed to digital SET and analog RESET switching characteristics. For the Ti/Ta_2_O_3−x_/Pt neuromorphic device, the Ti top and Pt bottom electrodes are considered as counterparts of the pre-synaptic input terminal and a post-synaptic output terminal, respectively.

## Introduction

Synapses are the most elegant connections in the brain, in which different kinds of electrical activities occur during the learning process of the brain. A synapse is two-terminal transmission line between pre-synaptic input (Axon firing spikes) and post-synaptic output (Dendrite receiving the transmitters) responding with the generation of post-synaptic potential. The logical calculus is immanent in the nervous activity, because of its “all-or-none” characteristics^1^. The synaptic weight of the synapse can be adjusted by the ionic (e.g. Ca^+2^) flow through them because of the action potentials, and this phenomenon is thought to be the cause for the process of learning and forgetting function in the brain^2–5^.

A memristor based neuromorphic device has resemblance with the synapse due to its two terminal structure, sandwiched insulator and continuous conductance change. Therefore, a memristor can exhibit as a neuromorphic device which can demonstrate the learning rule of the synapse, such as spike-timing-dependent plasticity (STDP)^6–8^. A neuromorphic device must emulate the synaptic plasticity, which is the ability to modulate synaptic weight. The synaptic plasticity includes the learning/forgetting, STDP and spike-rate-dependent plasticity (SRDP)^4,9^. The behavioral change caused by an experience is called memory and the process of acquiring memory is called learning. The nerve cells (neuron) communicate with each other via a memory storage candidate called synapse^1^. Current computer can be used to mimic the brain activity of cat and mouse^10–12^, but the power consumption increases exponentially with the animal intelligence.

CMOS coupled with multiple transistors^13,14^ are used in order to emulate the synapse in solid state device level. In addition, carbon nanotube (CNT) field effect transistors are also demonstrated for neuromorphic synaptic device^15^. However, these techniques limit the density of the memory as well as consume substantial power and therefore, are not feasible for human brain function device. On the other hands, two terminal memory devices like phase change memory^16^ and conductive bridge memory^7,17^ which are also types of resistive random access memories (RRAM)^18–21^ can be programmed with less power consumption for the neuromorphic applications. Therefore, the RRAM device is considered as the most suitable neuromorphic device candidate due to lower power consumption and CMOS process compatibility^7,20,22–24^. Digital resistive switching characteristics and multilevel resistive switching have been reported in many materials with very low power consumption^25–28^. The conductance of RRAM based analog switching devices changes for specific values of input pulses and this change in conductance is thought to be a counterpart of synaptic weight change of the synapse^7,29^. The switching characteristics of RRAM devices are usually classified into filamentary type^30,31^ and interface type^32–34^. Usually, RRAM devices are in a very high resistance state i.e., nearly a perfect insulator in their pristine state. Prior to any reliable and reproducible switching cycle one higher bias step is needed which is called electroforming or forming process^35–37^. The process of electroforming is indeed an undesirable power consuming and time-consuming step in order to measure the non-volatile characteristics of RRAM. For eliminating this undesirable electroforming process, many researchers have contributed to fabricate forming-free RRAM devices^38–40^. Another issue with RRAM is the compliance current which is usually needed to apply during electroforming and SET process in order to avoid a permanent or hard breakdown and transistors are being used as current compliance element^41,42^. Without compliance current devices may permanently damage and remain at the low resistance state (LRS) from where the device cannot be RESET to high resistance state (HRS). The compliance current requirement can limit the characteristics of the devices during the transient electrical measurements.

In order to use RRAM as the neuromorphic device, it must exhibit an incremental switching instead of a binary switching^7,29,43–46^. The overall efficiency of analog switching in neuromorphic application significantly depends on the role of the number of resistance levels, operational speed and power consumption plays^43,47,48^. RRAM has been demonstrated as neuromorphic device with different switching materials and processes such as WO_x_^43^, Si-doped TaO_x_^49^, multiple stacked HfO_x_/TiO_x_/HfO_x_/TiO_x_^50^ and SiO_x_/TaO_x_^44^. TaO_x_-based RRAM can be applicable as a neuromorphic device due to its highest endurance and reliability reported in literature^20^. For attaining suitable TaO_x_ switching layer as a neuromorphic device, Zongwei *et al*. engineered TaO_x_-based RRAM by inserting SiO_2_ layer as diffusion limiting layer (DLL) between TiN and TaO_x_ interface^44^. This DLL limits the ion diffusion speed and therefore, reduces the number of oxygen vacancies/ions that participate in the initial and abrupt filament growth/dissolution process. Thefore, the defect concentration difference at the interface of TiN and TaO_x_ can be reduced by insertion of the SiO_2_ DLL. Their report indicates a moderate defect concentration difference at the interface and switching layer plays a key role in rendering RRAM binary device to incremental switching or conductive neuromorphic device. The conductance modulation required for neuromorphic functionalities is also affected by transient electrical characteristics on the device. The increase and decrease in the conductance using programming pulses critically depends upon the rate of stimulation^21,51^. It is reported that the metal cations and O_2_ anions are mobile and participate in the resistive switching process. For the thin films of TaO_x_, HfO_x_ and TiO_x_ it is proved that the cations are mobile under the influence of electric field and can actively participate in the resistive switching process in competition with the oxygen vacancies^52,53^. In order to understand the effect of interlayers^54^ on the resistive switching in Ta/TaO_x_ devices the graphene layer is introduced at the Ta/TaO_x_ interface. Same results are also observed by introducing the amorphous carbon layer at Ta/TaO_x_ interface^55^. These interface layers result in the change of switching dynamic from valence change memory (VCM) to electrochemical metallization memory (ECM) mode. The moisture also affects the resistive switching characteristics of the RRAM device. Although we did not measure the effect of moisture on the electric characteristics of the device but the moisture effect on the memory characteristics are discussed in details in the literature^53,56^.

In this study, we fabricate and characterize a composition modulated tantalum oxide-based RRAM as a potential synaptic device. Both Ta_2_O_5_ and Ta targets are used to tune the composition of the deposited tantalum oxide (Ta_2_O_3−x_) by an *in-situ* co-sputtering. In particularly, a digital SET and analog RESET characteristics in the tantalum oxide-based memristor with compliance-free electroforming and SET processes is reported. This type of electrical characteristics with Ta/Ta_2_O_5_/Pt structure is reported by Wedig *et al*.^52^. For better understanding on this unique behavior, we propose an electrical switching model on the basis of oxygen vacancies (V_o_s) movement in the switching medium with comprehensive understanding of the DC and AC electrical characteristics on the devices. In addition, synaptic characteristics are demonstrated by applying appropriate voltages pulses. An abrupt change in conductance for positive pulse as well as continuous change in conductance for negative pulses implies the digital SET and analog RESET switching characteristics. Finally, we obtain the forgetting characteristics from the device using negative stimuli.

## Results and Discussion

**Figure 1:**
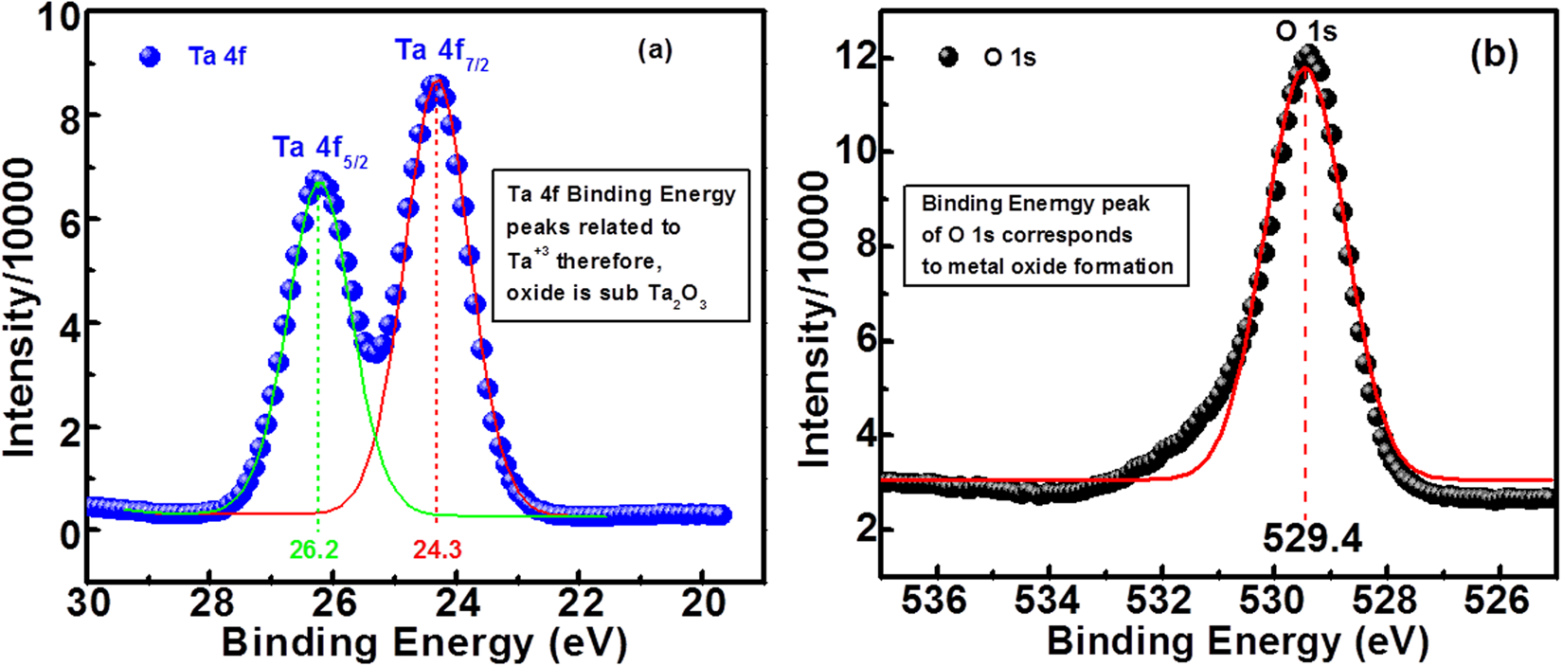
XPS spectra of (**a**) Ta 4 f and O 1 s within deposited tantalum oxide, indicating the formation of sub tantalum oxide (Ta_2_O_3−x_).

In order to investigate the chemical composition of the tantalum oxide thin film, the thin film is examined using x-ray photoelectron spectroscopy (XPS). Figure 1(a) and (b) shows the XPS BE peaks of Ta 4 f and O 1 s, respectively. From the peaks ratio of tantalum binding energy (B.E) in Fig. 1(a) which is 3:4, it is confirmed that Ta 4f_5/2_ and Ta 4f_7/2_ exists at the peak values of 26.2 eV and 24.3 eV, respectively. These peak values are related with Ta^+3^ as reported^57,58^ and confirms most of the sputtered thin film is composed of Ta_2_O_3−x_. Figure 1(b) shows the XPS BE peak of oxygen at 529.4 eV which depicts the existence of metal oxide (Ta_2_O_3_ in this study).

**Figure 2:**
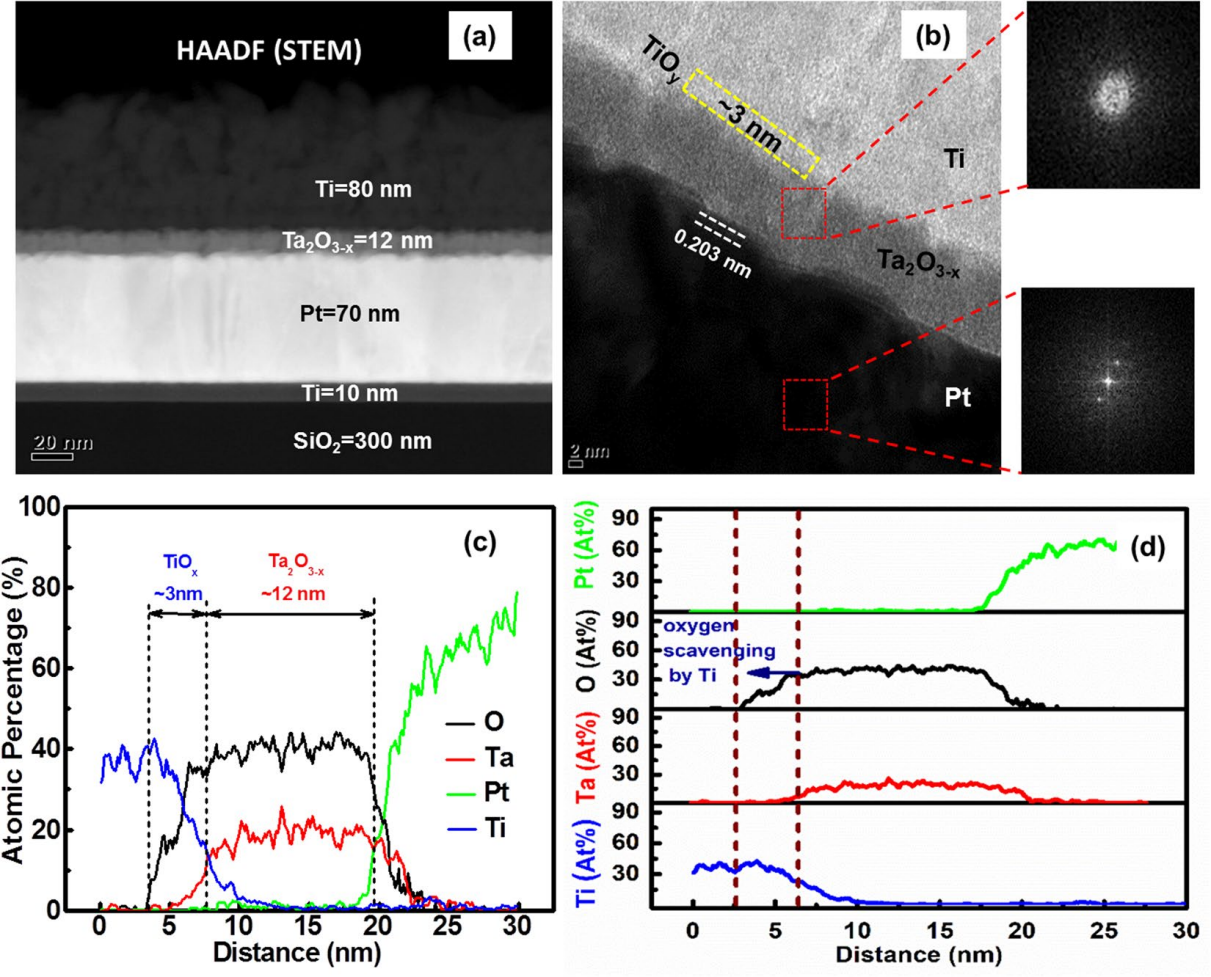
(**a**) STEM HAADF image of Ti/Ta_2_O_3−x_/Pt RRAM device, (**b**) high resolution STEM bright field image of the device with FFT (STEM). Red squares indicate the amorphous and crystalline nature of Ta_2_O_3−x_ and Pt, respectively, (**c**) EDX profiles of the all elements within Ti/Ta_2_O_3−x_/Pt structure showing the thickness of Ta_2_O_3−x_ thin film and (**d**) individual line profiles of every elements of the device along the vertical direction.

Figure 2 shows the physical characteristics of tantalum oxide based neuromorphic device investigated using transmission electron microscopy (TEM) and energy-dispersive x-ray spectroscopy (EDX) analysis. Figure 2(a) shows the cross-sectional scanning TEM (STEM) high angel annular dark field (HAADF) image of the fabricated device. Figure 2(b) shows high resolution STEM bright field image of the devices with FFT (TEM) images, where the amorphous nature of Ta_2_O_3−x_ thin film and crystalline nature of Pt are clearly shown within the red square regions, respectively. The amorphous nature of Ta_2_O_3−x_ film infers the presence of oxygen vacancies (V_o_s)/defects inside the thin film. The different contrast within yellow rectangular region at the interface of Ti/Ta_2_O_3−x_ is due to the formation of oxygen deficient titanium oxide (TiO_y_) thin layer with 3 nm thickness. Compared to our previous result on the interface between Ti and Ta_2_O_5_ films^59^, interface thickness of Ti/Ta_2_O_3−x_ increases by ~1.5 nm. This thickness increase is attributedthat Ti top electrode having high Gibbs free energy of oxide formation (−888.8 KJ/mole) is more favorable to scavenge oxygen from sub-tantalum oxide (Ta_2_O_3−x_) than that from stable Ta_2_O_5_, leading to thicker TiO_y_ formation. This comparatively thicker layer of TiO_y_ films offers the digital SET and analog RESET electrical characteristics in the device. We speculate that this TiO_y_ layer is favorable for the unique digital SET and analog RESET switching observed from the electrical characterization of our Ta_2_O_3−x_ based RRAM device. This TiO_y_ thickness is expected to be modulated by varying an *in-situ* co-sputtering of Ta_2_O_3_ and Ta process condition. Figure 2(c) exhibits the collective intensity profiles of Ti, Ta, O, and Pt along the vertical direction and thickness of Ta_2_O_3−x_ in Ti/Ta_2_O_3−x_/Pt structure. Figure 2(d) shows the individual intensities of elements along the vertical direction of the device. These oxygen profiles clearly show the existence of oxygen in at the interface, correlating to the formation of TiO_y_ at the interface. Hence, the interface between Ti and Ta_2_O_3−x_ is the reservoir for the oxygen vacancies (V_o_s) and the device structure can be considered as Ti/TiO_y_/Ta_2_O_3−x_/Pt. To investigate the synaptic transmission characteristics of tantalum oxide based neuromorphic device an electroforming step is needed without any compliance current (compliance-free) in this study.

**Figure 3:**
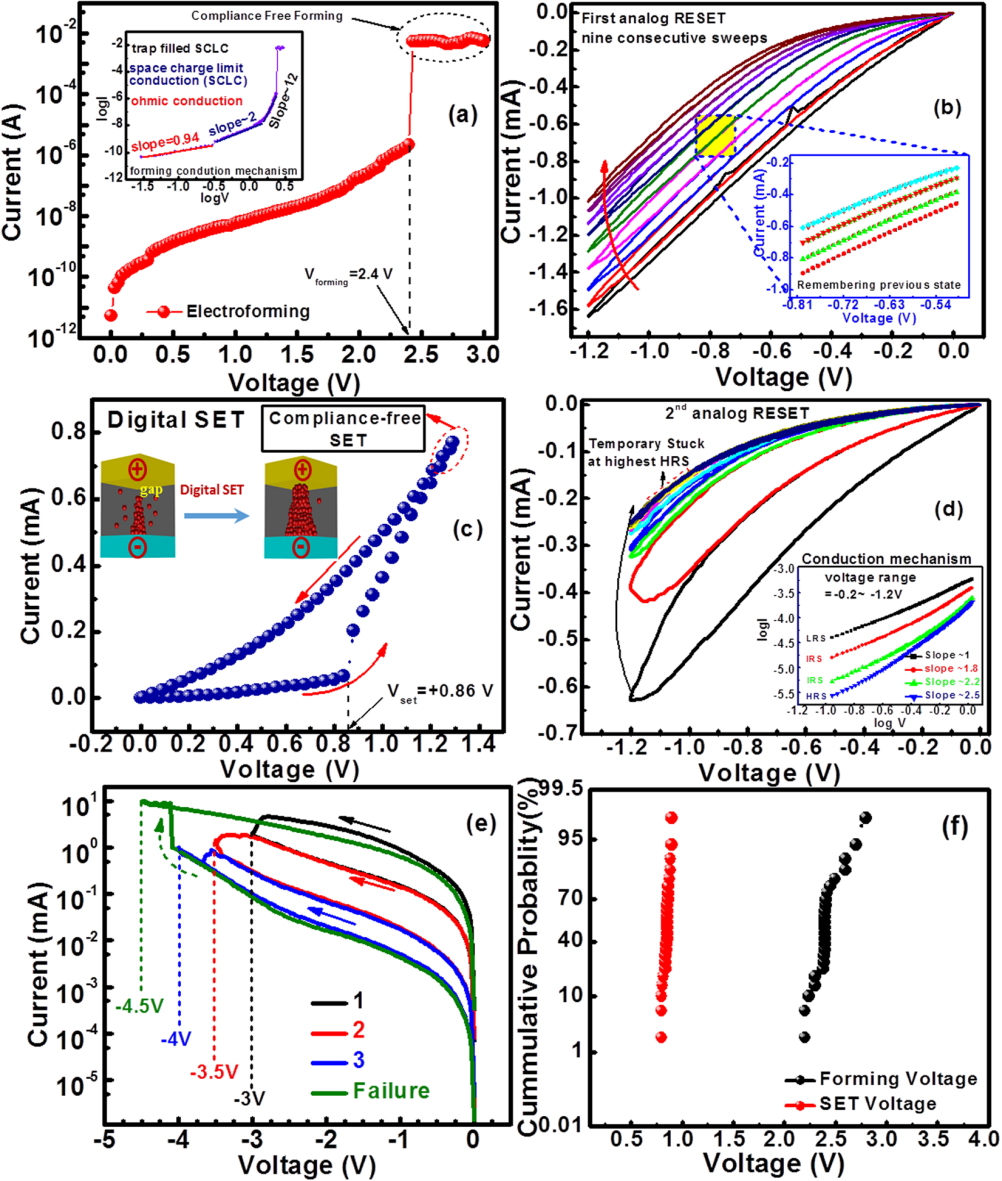
The electrical responses of Ta_2_O_3−x_-based neuromorphic device under various measurement conditions with (**a**) the compliance-free electroforming of Ta_2_O_3−x_ based neuromorphic device and inset showing the conduction mechanism during forming, (**b**) analog RESET process of the device under the consecutive nine sweeps using −1.2 V, (**c**) the digital compliance-free SET process with double sweeps in the range of 0 ~ +1.3 V, (**d**) analog RESET with double sweeps in the range of 0 ~ −1.2 V showing the temporary stuck at the highest HRS, (**e**) analog switching behaviors using higher voltages with gradual voltage increase in magnitude at the negative bias and it leads to final failure process, and (**f**) cumulative probability graphs of forming voltage and SET voltages.

Figure 3 shows the electrical responses of Ta_2_O_3−x_-based neuromorphic device under various measurement conditions. Figure 3(a) represents the forming process by applying a bias voltage sweep of +3 V where the forming voltage is +2.4 V. Even though the exact location of the formed filaments inside the switching medium is unpredictable^60^, as discussed previously that the resistance levels of the off-state device and HRS at the reading voltage of 0.5 V are 6.8 × 10^8^ Ω and 1.9 × 10^4^ Ω, respectively, which evidences the formation of filament in our devices. In order to bring the device into its highest HRS we applied the consecutive DC sweeps of voltages −1.2 V as shown in Fig. 3(b). Inset shows the device remembers its previous state excellently during each negative sweep for every intermediate resistance states (IRS). After achieving highest HRS with consecutive 9 voltage sweeps of −1.2 V, the device current level becomes temporarily stuck at the highest HRS. These results show that the repetitive application of negative bias sweep can decrease the conductance of Ta_2_O_3−x_ memristor. To increase the conductance of the device in the analog fashion we applied positive voltage sweeps under different voltage sweep ranges (not shown here), but did not observe any variation in highest HRS level. At a specific voltage sweep of +1.3 V, a digital or abrupt SET is observed at the SET voltage of VSET = +0.86 V, manifesting the conversion from an analog RESET to digital SET state as shown in Fig. 3(b) and (c).

These conductance modulations of Ta_2_O_3−x_ neuromorphic device for negative sweeps correspond to the nonlinear behavior observed in the biological synapse^61,62^. Figure. 3(d) confirms the temporary stuck of the current level at highest HRS state. Once device reaches to highest HRS state, then the next negative sweeps of −1.2 V do not change the resistance level of the device from highest HRS. The inset shows the conduction mechanism from LRS to IRS to HRS levels at the voltage range of −0.2 V ~ −1.2 V. Before an electroforming process, the resistance of the off state device (before electroforming) and the resistance in HRS state at the reading voltage of 0.5 V is calculated from the Fig. 3(a) and (c) as 6.8 × 10^8^ Ω and 1.9 × 10^4^ Ω, respectively. The number of sweeps applied during analog RESET process before the resistance level reaches to highest HRS is observed to be nearly dependent on the values of the negative sweep voltage, i.e., higher the sweep voltages smaller the number of resistance states and vice versa^50^. In order to prove this statement, the negative sweeps with higher voltage values are applied such that every sweep voltage has slightly higher voltage value as compared to its previous value i.e. −3 V, −3.5 V, −4 V and −4.5 V. It is observed that the device damaged by permanently stuck to LRS state at a specific highest value (−4.5 V) of the sweep as shown in Fig. 3(e). Hence, from Fig. 3(e) we conclude that the device exhibits digital SET and analog RESET switching and the number of sweeps during analog switching depend on the values of the negative sweeps. In order to investigate some randomness or reliability in the forming process and SET process we measured 50 different individual devices. The variation in forming voltages and SET voltages were not very high as can be seen from the cumulative probability graphs in Fig. 3(f). For the negative sweep voltages of less than −1 V, the devices remained at their LRS and further increased of voltage sweeps (from 0 V → −1.2 V → 0 V and 0 → −3.5 V → 0 V) the analog reset is observed. When the negative sweep of 0 V → −4 V → 0 V is applied an abrupt SET is observed rendering device to permanent or hard breakdown as shown in Fig. 3(e). For the analog RESET cycles using 0 V → −1.2 V → 0 V nine different and distinguishable states are observed.

**Figure 4:**
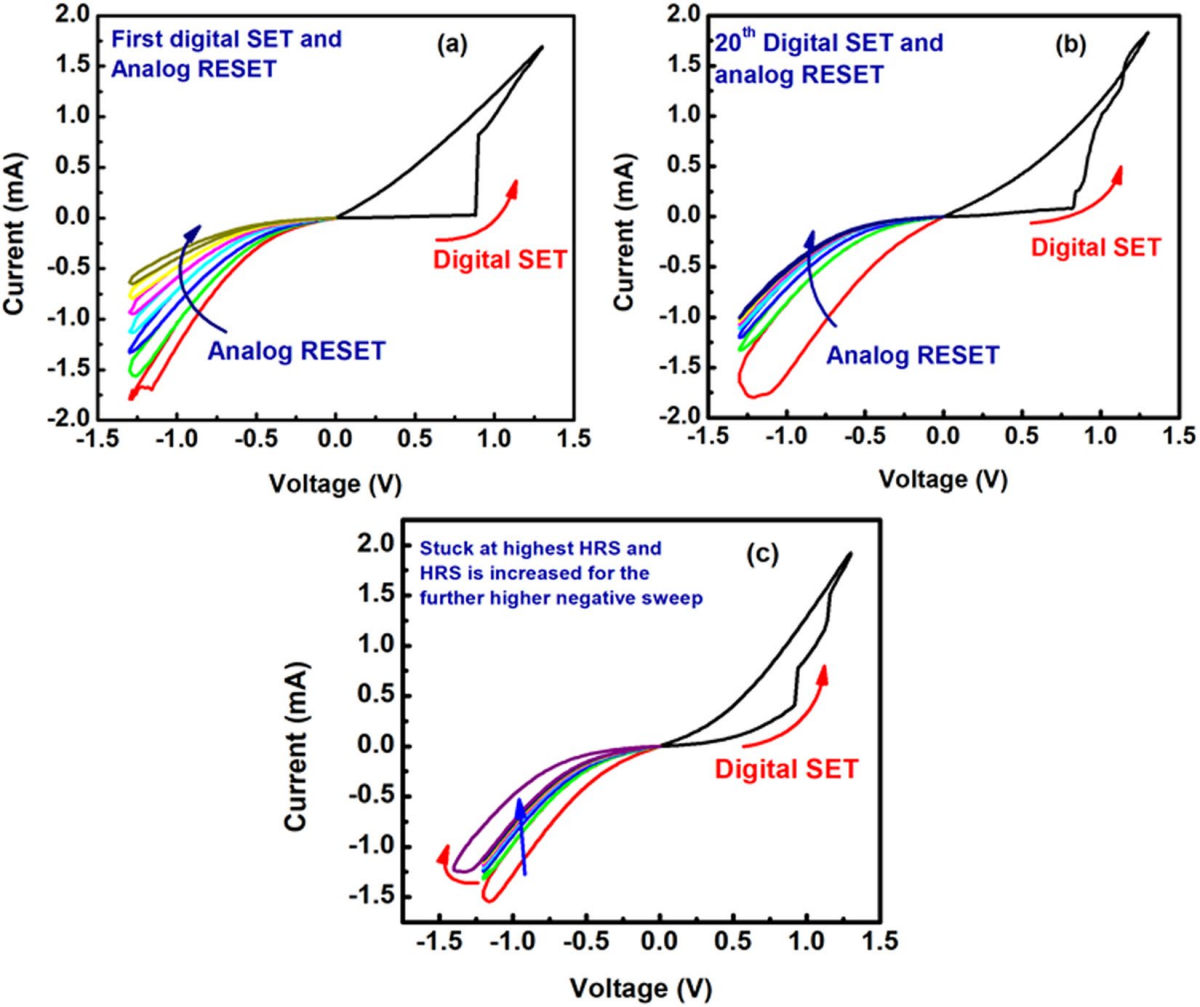
The repeatability test for consecutive digital SET and analog RESET of the 1^st^ digital SET and analog RESET (**a**) as well as the 20^th^ digital SET and analog RESET (**b**). The (**c**) is the increase of HRS for the higher value of voltage sweep.

Figure 4(a) and (b) show the possible degradation in the repeatability of the switching in the device after 1^st^ through 20^th^ digital SET and analog RESET cycles, respectively. It is observed that the first analog RESET sweeps are not exactly same as that of the 20^th^ analog RESET (0 V→ −1.4 V → 0 V). It is clear that the device works for the consecutive digital SET and analog RESET cycles, but the current level slightly increases at the highest HRS of the 20^th^ cycle as compared to that of 1^st^ cycle. We also observed that once the resistance level stuck with the highest HRS for the application of constant sweep, the resistance of the device can be further reduced by applying a suitable higher sweep voltage. In order to realize this statement, during the digital SET and analog RESET cycles we did analog RESET cycles intentionally for the lower voltage of −1.2 V and observed that device stuck to its highest HRS. After the device resistance stuck with highest HRS we applied slightly higher voltage of −1.4 V which again increased the resistance level of the devices as shown in Fig. 4(c).

**Figure 5:**
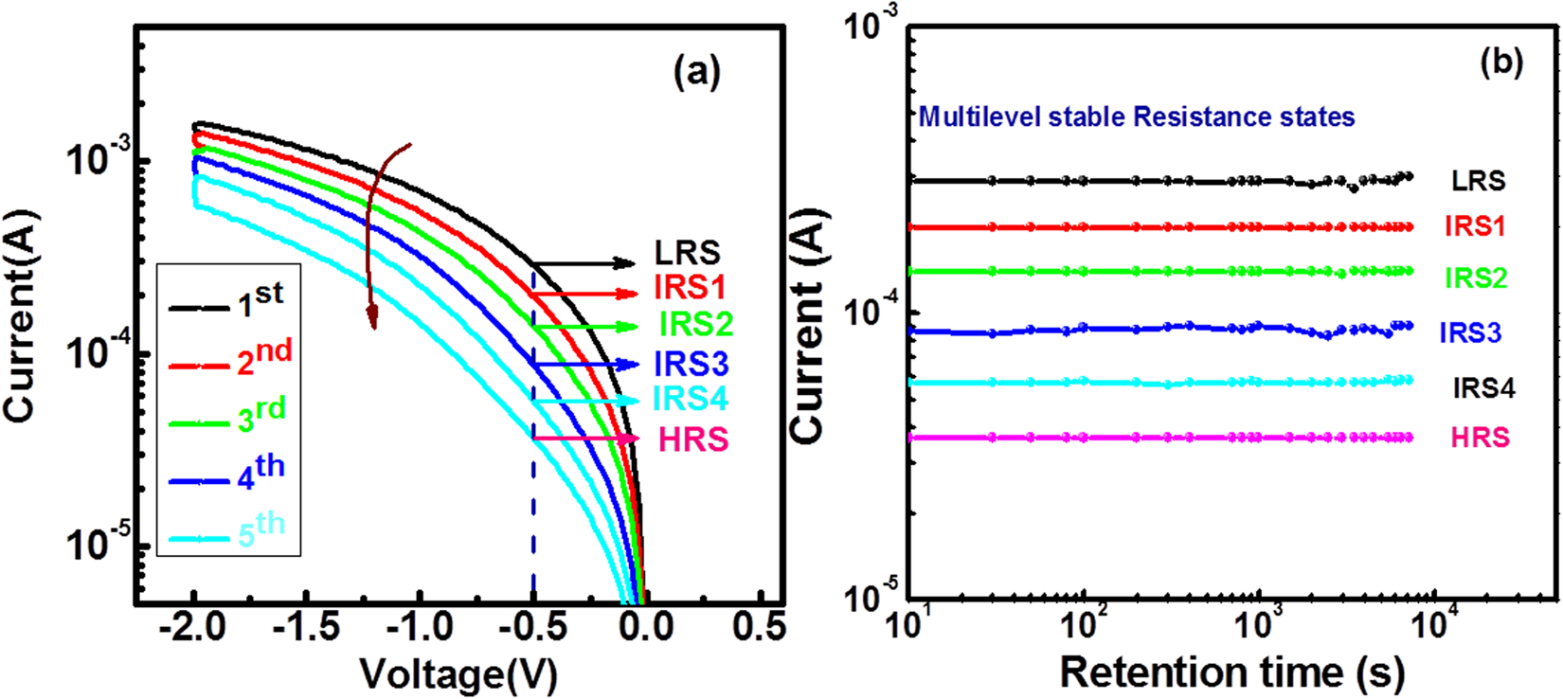
The retention characteristics of device (**a**) The analog RESET sweeps (**b**) the stability of LRS, IRS and HRS at the reading voltage of −0.5 V.

For the confirmation of each resistance level stability with time during analog RESET sweeps we measured the retention of each state of the device at the reading voltage of −0.5 V for the time of 7.2 × 10^3^ sec. The LRS, intermediate resistance states (IRS 1, IRS 2, IRS 3, and IRS 4) and HRS were stable with time as shown in Fig. 5. The retention characteristics of each resistance level were measured during analog RESET using voltage sweeps of 0 V→ −2 V → 0 V. Figure 5(a) shows the analog RESET and Fig. 5(b) shows the retention of the corresponding resistance states at the reading voltage of −0.5 V.

The switching type could be interface or filamentary depending on the device structure and electrical characteristics exhibited by the device. Since for the present device the electroforming step is required to obtain the further digital SET and analog RESET, the device falls into the filamentary typed RRAM device. For the present work we propose the switching mechanism of the device or resistance modulation is based on the moment of V_o_s or oxygen ion migration with the redox reaction process, which results in the formation/partial dissolution of the conduction filament (CF) formed by V_o_s-rich region inside the switching layer during the SET and analog RESET process^23,63–65^. The TiO_y_ layer is formed at the interface of Ti/Ta_2_O_3−x_/Pt and this interface layer is solely responsible for the resistive switching (after forming process) and allows the movement of oxygen vacancies as well as electrons. The formation of filament can be explained using defect chemistry. Since TiO_y_ is a hypostoichiometric transition metal oxide, the hypostoichiometry results from the formation of cation interstitials or oxygen vacancies. The formation of oxygen vacancies and cation interstitials can be expressed in Kröger-Vink notation as;

1 $${{\rm{O}}}_{{\rm{o}}}^{{\rm{x}}}\to {{\rm{V}}}_{{\rm{o}}}^{{\boldsymbol{\cdot }}{\boldsymbol{\cdot }}}+2{{\rm{e}}}^{-}+{1/2}{{\rm{O}}}_{2}({\rm{g}})$$

For the formation of metal interstitials we can write the following notation;

2 $${{\rm{M}}}_{{\rm{M}}}^{{\rm{x}}}+{{\rm{O}}}_{{\rm{o}}}^{{\rm{X}}}\to {{\rm{M}}}_{{i}}^{{\boldsymbol{\cdot }}{\boldsymbol{\cdot }}}+2{{\rm{e}}}^{-}+{1/2}{{\rm{O}}}_{2}({\rm{g}})$$

In case of Ti we can write as;

3 $${{\rm{Ti}}}_{{\rm{Ti}}}^{{\rm{x}}}+{{\rm{O}}}_{{\rm{O}}}^{{\rm{X}}}\to {{\rm{Ti}}}_{{i}}^{{\boldsymbol{\cdot }}{\boldsymbol{\cdot }}}+2{{\rm{e}}}^{-}+{1/2}{{\rm{O}}}_{2}({\rm{g}})$$

where positive charge is represented by a dot (•), and neutral by (×). The subscript represents defect site (*i*) for interstitial, and (Ti) for titanium lattice site.

**Figure 6:**
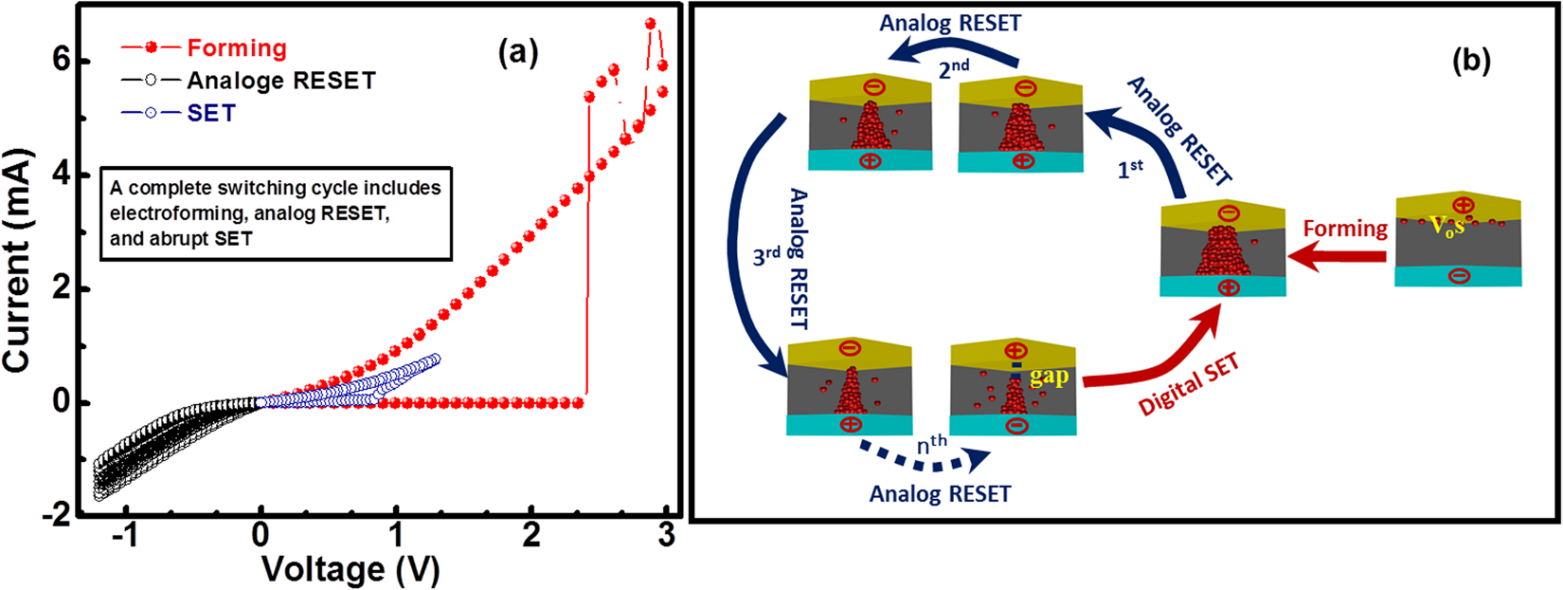
Switching mechanism of neuromorphic memristor shows (**a**) the process of forming, analog RESET and SET as well as (**b**) the proposed switching mechanism.

Based on digital SET and analog RESET switching observation, we hypothesize that the filament formation (SET) is the abrupt process, whereas the gradual RESET follows the partial dissolution of the filament during each negative cycles until filament breaks and an insulating gap is formed between the remaining filament and the top electrode. The switching mechanism is explained in Fig. 6. This process of electroforming, analog RESET and then abrupt SET is represented in Fig. 6(a).

During the analog RESET we observe that the device remembers its previous resistance state until it reaches to its highest HRS as shown in Fig. 3(b) and (d) and does not observe any overlapping of RESET sweep with its previous sweeps as reported in^66,67^. Hence, device exhibits an excellent digital SET and analog RESET switching memory characteristics. The switching mechanism for the digital SET and analog RESET is shown schematically in Fig. 6(b).

**Figure 7:**
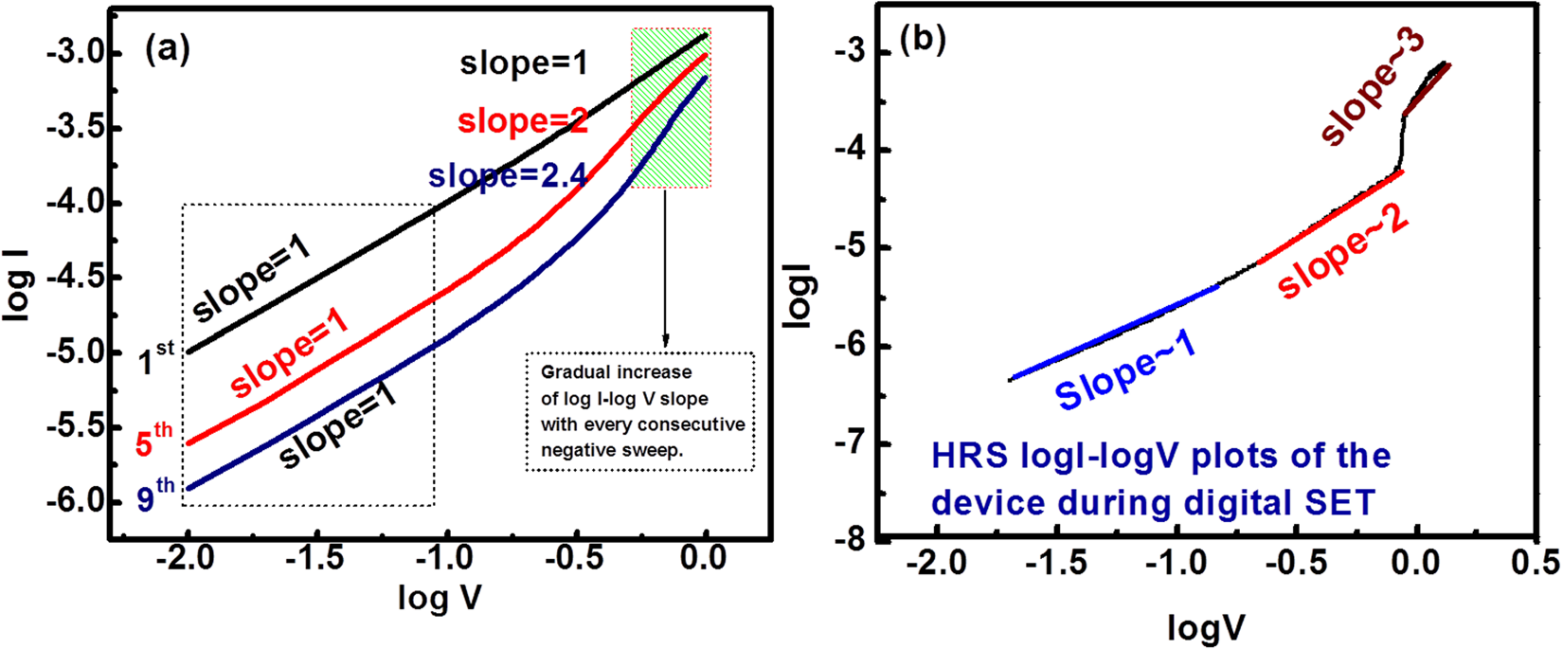
Conduction mechanism of the device for analog RESET and digital SET (**a**) Log I-Log V plot of 1^st^, 5^th^ and 9^th^ sweep from the consecutive sweep of analog RESET and (**b**) LogI-LogV plot of the SET process

Figure 7 shows log I vs. log V plot in order to confirm our speculation on the switching mechanism, where we replotted 1^st^, 5^th^ and 9^th^ sweeps of the analog RESET of Fig. 3(b). It is observed that at lower voltages slopes of all sweeps are equal to 1, indicating the ohmic conduction whereas for higher voltages (−0.8 V to −1.2 V) the slopes of 1^st^, 5^th^ and 9^th^ sweeps are 1, 2 and 2.4, respectively. For the 1^st^ negative sweep, slope at all voltage range is clearly equal to 1 indicating ohmic conduction while for 5^th^ sweep slope at higher voltage is equal to 2, infers the space charge conduction limited (SCLC) i.e. IαV^2^ and finally for the 9^th^ sweep the slope of 2.4 at higher voltages confirms the trap filled SCLC i.e. IαV^a^ where a > 2. In order to discuss the conduction mechanism during digital SET we replotted Fig. 3(c) in logI-logV scale as shown in Fig. 7(b). It can be seen at lower voltages the conduction is ohmic and for higher voltages conduction is SCLC as well as trapped filled SCLC.

This change in nature of I-V characteristics at higher voltages from Ohmic to SCLC to trap filled SCLC during the consecutive applications of negative sweep stimulus indicates the filament dissolution in the analog RESET processes which is in accordance with our hypothesized switching mechanism.

**Figure 8:**
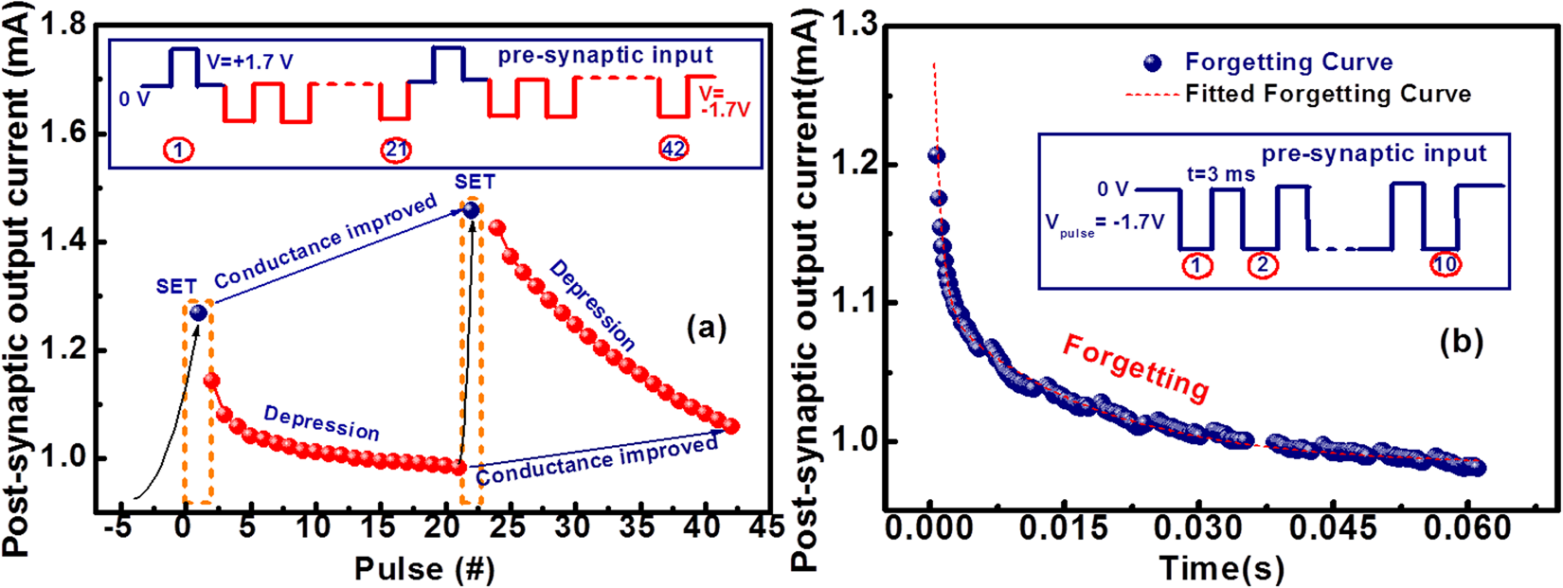
The synaptic characteristics of Ta_2_O_3−x_ memristor device show (**a**) the electrical responses for the programming pulses exhibiting the digital and gradual decrease of the conductance upon the negative pulses (depression process) with pre-synaptic input pulse condition and (**b**) the forgetting curve of the memristor based neuromorphic device fitted with equation (1).

Figure 8 shows the synaptic characteristics of Ta_2_O_3−x_ based memristor device. The post-synaptic output current behavior upon programming pulses is shown in Fig. 8(a). To investigate synaptic plasticity, the train of pre-synaptic input (voltage pulses) is applied by keeping initially the device in HRS. One positive pulse (amplitude = 1.7 V and duration = 3 ms) followed by twenty negative pulses (amplitude = −1.7 V and pulse width = 3 ms) and then repeated the above sequence as shown in the inset of Fig. 8(a). During this transient measurement, post-synaptic currents (the output current or conductance) of the memristor are monitored and it is observed that the positive pre-synaptic pulse caused an abrupt change in synaptic weight (conductance) and negative pulses decreased the current of the device gradually as shown in Fig. 8(a). This phenomenon is the best emulation of the synaptic plasticity. It is observed that the conductance of device is improved at the 42^nd^ pulse as compared to the 21^st^ pulse. The decay of synaptic weight of Ta_2_O_3−x_ memristor emulated the memory forgetting curve of human brain which is given by the forgetting curve, and is given by following equation^65,67–69^.

4 $$W(t)={W}_{e}+A\cdot exp(-t/\tau )$$

where *W(t)*, *W*_*e*_, *A* and *τ* are the synaptic weight of device at time *t*, synaptic weight at stable state, constant and relaxation time constant, respectively.

Figure 8(b) shows the forgetting characteristics with the pre-synaptic input pulses. After keeping the neuromorphic device in LRS we measured the forgetting curve of the device. In order to obtain forgetting curve, we applied ten pre-synaptic input pulses (V = −1.7 V, pulse width = 3 ms) and post-synaptic current in monitored with time. The forgetting curve of the device nearly fits with the forgetting curve of the human brain of equation (4).

## Conclusion

A neuromorphic device with Ta_2_O_3−x_ has been investigated to emulate the function of the synapse in the brain. A digital SET and analog RESET switching behavior is observed with this type of device under the appropriate positive sweep and consecutive negative sweeps, respectively. The device exhibits a compliance-free electroforming and SET processes. An abrupt SET and gradual RESET (analog) characteristics are observed for the positive sweep at V_set_ = +0.86 V and for the multiple consecutive negative sweeps of V_reset_ = −1.2 V, respectively. This unique switching observation is attributed to the oxygen vacancies moment we proposed. Finally, we demonstrate synaptic plasticity and forgetting curves upon suitable pulse stimuli, confirming the emulation of memristor-based neuromorphic device synaptic plasticity and forgetting function of the brain. Our approach suggests that devices with excellent memory characteristics can be attained without overlapping of resistance level with its previous states.

## Method

### Device Fabrication

The tantalum oxide-based neuromorphic device was fabricated by depositing ~12 nm of Ta_2_O_3−x_ thin film on the Pt/Ti/SiO_2_ substrate. Platinum (Pt) layer acts as a bottom electrode. The Ta_2_O_3−x_ thin film was formed by the *in-situ* co-sputtering process with Ta_2_O_5_ ceramic target and Ta target under the Ar ambient at the working pressure of 4 mTorr and Ar flow of 40 sccm. Then, ~80 nm thick Ti metal was sputtered as a top electrode. A 10^4^ μm^2^-sized device was finally fabricated with a lift-off process, where the final structure is Ti (80 nm)/Ta_2_O_3−x_ (12 nm)/Pt.

### Physical Characterization

Transmission electron microscope (TEM) sample was prepared using a focus ion beam system of FEI system. A milling voltage of 30 KV and current of 7 × 10^−9^ A was used during the milling of the sample using Ga ion. A probe-corrected JEOL 2100 F TEM was used for the study of a structural and stoichiometric characteristic of the device. Using scanning mode of TEM (STEM) a fast Fourier transform (FFT) image was obtained in order to confirm the amorphous and crystalline nature of thin films. An accelerating voltage of 200 KV was used during the TEM analysis. X-ray photoelectron spectroscopy (XPS) measurement was carried out on the Ta_2_O_3−x_/Pt/Ti/SiO_2_ stacks using theta probe of thermo scientific company. A monochromatic Al Kα X-ray gun was used with the energy of 15 KeV.

### Electrical Characterization

A Keithley 4200 semiconductor characterization analyzer was used to study the DC and transient electrical characteristic of the neuromorphic device. During all electrical measurements, the biasing voltage was applied to the top electrode (TE) while bottom electrode (BE) was kept grounded.
